# Fosfomycin combination therapy in high-risk paediatric patients with multidrug-resistant infections: a retrospective study

**DOI:** 10.1007/s00431-026-06822-9

**Published:** 2026-04-10

**Authors:** Marco Denina, Laura Badiali, Raffaele Vitale, Francesca Robasto, Elisa Funiciello, Erika Silvestro, Giulia Pruccoli, Silvia Garazzino

**Affiliations:** 1 Department of Pediatrics, Infectious Diseases Unit, University of Turin, Regina Margherita Children’s Hospital, Turin, Italy; 2 Department of Pediatric Emergency, Regina Margherita Children’s Hospital, Turin, Italy; 3 Department of Pediatrics, Ospedale di Chivasso, 10034 Chivasso, Italy; 4 Department of Pediatrics, Ospedale Civile Giuseppe Mazzini, 64100 Teramo, Italy; 5 Department of Pediatric Hematology and Oncology, Regina Margherita Children’s Hospital, Turin, Italy

**Keywords:** Antimicrobial stewardship, Combination therapy, Intravenous fosfomycin, Multidrug-resistant infections, Paediatric high-risk patients, Safety profi le

## Abstract

Intravenous fosfomycin is a broad-spectrum, bactericidal antibiotic active against Gram-positive and Gram-negative bacteria, including multidrug-resistant (MDR) strains. Evidence regarding its use in paediatric patients, particularly as part of combination therapy, remains limited. This study aimed to evaluate the safety and clinical outcomes of intravenous fosfomycin-based combination therapy in children. We conducted a single-centre, retrospective study including paediatric patients aged 0–18 years who received IV fosfomycin at Regina Margherita Children’s Hospital in Turin between July 2019 and April 2025. Patients with underlying comorbidities, immunosuppression, invasive devices, or prematurity were included. Fifty paediatric patients were enrolled, with a median age of 5.93 years and balanced sex distribution. Comorbidities were present in 80% of cases, and 56% had primary or acquired immunodeficiency. Fosfomycin was always used in combination therapy for confirmed bloodstream infections (54%), osteoarticular infections (12%), skin and soft tissue infections (8%), or other infections (24%). Microbiological identification was achieved in 90% of cases: 53.4% *Gram*-negative and 46.6% *Gram*-positive bacteria. Half of the isolated pathogens were MDR, including ESBL, CPE, MDR *Pseudomonas aeruginosa*, XDR and MDR *Gram*-positive bacteria. Five patients died, and therapeutic failure—defined as death, relapse, or failure to achieve microbiological clearance—occurred in 28% of cases. IV fosfomycin was well tolerated, and no adverse effects requiring treatment discontinuation were reported.

*Conclusions*: In a cohort of vulnerable paediatric patients with severe underlying conditions and difficult-to-treat infections, IV fosfomycin-based combination therapy was well tolerated and associated with acceptable clinical outcomes. Its use should be considered a rescue therapy for infections resistant to standard treatment.

**What is Known:**

• *Fosfomycin is an old antibiotic that has been recently re-evaluated for intravenous use in severe infections causedby multidrug-resistant pathogens*.

• *It is increasingly used in combination regimens for diffi cult-to-treat infections, but paediatric evidence remainslimited*.

**What is New:**

• *In a real-world cohort of high-risk children, fosfomycin-based combination therapy was associated with favourableclinical responses in infections due to MDR bacteria*.

• *Treatment was generally well tolerated, with limited clinically significant adverse events, even in a fragile andimmunocompromised paediatric population*.

## Introduction

Fosfomycin is an antibiotic discovered in 1969, initially derived from the fermentation of Streptomyces. It is a bactericidal agent, unique within the epoxide class, and it exerts its action by inhibiting bacterial cell wall synthesis through the inactivation of the enzyme UDP-N-acetylglucosamine-3-enolpyruvyltransferase, which is responsible for peptidoglycan cell wall synthesis. Due to its distinctive mechanism of action, cross-resistance to other classes of antibiotics is rare [1]. Moreover, when used in combination therapies, it has a synergistic effect, enhancing the antimicrobial and bactericidal activity of other drugs. Fosfomycin demonstrates a broad spectrum of activity, effective against both *Gram*-negative and *Gram*-positive bacteria, including multidrug-resistant (MDR) strains. Its synergistic activity has led to its increasing use in combination regimens for the treatment of infections caused by difficult-to-treat pathogens, such as *Pseudomonas aeruginosa* and *Acinetobacter baumannii*, for which fosfomycin is usually administered as part of multidrug regimens and has shown in vitro synergistic effects with other antimicrobial agents [2]. Fosfomycin is a small molecule with low protein binding, offering offering good tissue distribution and the ability to penetrate biofilms, achieving high concentrations even in tissues that are typically difficult to reach. In addition, fosfomycin remains active under low pH conditions, such as those found in abscesses. Traditionally, fosfomycin has been used orally to treat urinary tract infections (UTIs). However, its properties make it an effective intravenous therapy for a broad range of infections, including osteoarticular infections, central nervous system (CNS) infections, skin and soft tissue infections (SSTIs), pulmonary parenchymal infections, and sepsis [3]. Despite its promising profile, studies on the use of intravenous (IV) fosfomycin in paediatric populations remain limited [4–12]. This study aims to evaluate the tolerability and effectiveness of IV fosfomycin in combination therapies for treating severe infections in a complex paediatric population in a third-level university hospital in Turin, North-west Italy.

## Materials and methods

### Study design

This retrospective cohort study was a single-centre, descriptive analysis conducted at Regina Margherita Hospital in Turin, Italy. It included paediatric patients who received IV fosfomycin for different types of infections between July 2019 and April 2025. Data were retrospectively extracted from hospital records, and written informed consent was obtained in accordance with ethical guidelines.

### Aim of the study

This study aims to evaluate the tolerability and clinical outcomes of intravenous fosfomycin-based combination therapy in paediatric patients with complex infections. The analysis focuses on the dual challenge posed by highly resistant pathogens and the clinical fragility of the host. The outcomes include both safety, focusing primarily on the risk of electrolyte imbalances, and effectiveness, evaluated using a composite endpoint that includes mortality, lack of clinical improvement, relapse of infection or failure to achieve microbiological clearance by the end of the fosfomycin treatment course.

Intravenous fosfomycin was always administered as part of combination regimens and never as monotherapy. The rationale for its use included limited therapeutic options due to multidrug resistance, elevated MICs to first-line or novel β-lactams, the need for additional bactericidal activity in critically ill patients, and contraindications to aminoglycosides in the presence of renal impairment. This study therefore reflects real-world clinical practice rather than a predefined prospective treatment strategy.

### Inclusion criteria

The study cohort was composed of paediatric patients ranging from birth to 18 years of age. It was a heterogeneous group, comprising both previously healthy individuals and those with underlying comorbidities. Comorbid conditions included congenital heart disease (in some cases associated with syndromic conditions such as Noonan syndrome, Goldenhar syndrome, and Floating Harbor syndrome), cancers, chronic intestinal diseases (including Crohn’s disease and intestinal insufficiency), congenital malformations (e.g. severe scoliosis), cystic fibrosis, neurological disorders (e.g. spastic tetraparesis), and genetic syndromes (e.g. Loeys-Dietz syndrome). Premature newborns with severe invasive infections caused by MDR pathogens were also included. The study included patients with immunosuppression, either due to their underlying conditions or ongoing treatments, as well as those with invasive devices such as central venous catheters (CVC), endotracheal tubes (ET), extracorporeal membrane oxygenation (ECMO) circuits, surgical drainage devices, and percutaneous endoscopic gastrostomy (PEG) tubes.

### Infection types and treatment regimen

The study incorporated a broad range of infections, with a particular focus on confirmed sepsis, osteoarticular infections, skin and soft tissue infections (SSTIs), pneumonia, CNS infections and intra-abdominal infections. Patients were treated with different combination therapy schemes, which included fosfomycin IV, depending on the type of pathogen responsible, the previous systemic therapy and the type of infection.

### Microbiological data

Microbiological data were collected from blood cultures, site-specific cultures and surveillance swabs. Surveillance swabs were collected only in high-risk units (intensive care unit, oncology, and transplant wards) as part of routine infection control and antimicrobial stewardship programmes, to detect colonisation with multidrug-resistant pathogens and to support empirical treatment decisions in case of suspected infection. Positive cultures were classified as single-pathogen or polymicrobial infections. Pathogens were categorised based on *Gram* staining and further subclassified by species. The antibiograms of isolated pathogens were assessed to identify organisms susceptible to first-line antibiotics and those exhibiting resistance. Specifically, we focused on multidrug-resistant organisms, including extended-spectrum beta-lactamase (ESBL)-producing Enterobacterales, carbapenem-resistant Enterobacterales (CRE), multidrug-resistant *Pseudomonas aeruginosa* and oxacillin-resistant *Gram*-positive bacteria. Fosfomycin susceptibility was analysed for each pathogen, with minimum inhibitory concentrations (MICs) recorded, according to the European Committee on Antimicrobial Susceptibility Testing (EUCAST) [13].

### Therapeutic dose and tolerability of fosfomycin

The dose of fosfomycin was weight-based (Table 1) [1, 4, 7]. The total duration of therapy and the suspected or proven fosfomycin-associated side effects were recorded.

**Table 1:** Recommended paediatric dosing of intravenous fosfomycin according to age and body weight

Age	Dose
Preterm infants	100 mg/kg/day in 2 doses
Full-term infants	200 mg/kg/day in 3 doses
Age 1–12 months (< 10 kg)	300 mg/kg/day in 3 doses
Age 1–12 years (10–40 kg)	200–400 mg/kg/day in 3–4 doses
Age > 12 years (> 40 kg)	12–24 g/day in 2–3 doses

Fosfomycin susceptibility testing was routinely performed using the MicroScan automated system (Beckman Coulter). In selected isolates and upon specific request, minimum inhibitory concentrations were additionally determined by the reference agar dilution method in accordance with EUCAST recommendations.

To assess the tolerability and side effects of fosfomycin therapy, laboratory data routinely collected at the beginning and at the end of treatment were retrospectively analysed. These tests included complete blood count, liver function tests, kidney function tests, and electrolyte levels, performed as part of standard clinical care, to identify potential treatment-related alterations [7]. In addition, all abnormal laboratory values recorded during the treatment period were reviewed and analysed to identify potential treatment-related abnormalities or toxicities. The diagnostic blood values for drug toxicity were Grade ≥ 3 hepatotoxicity (AST/ALT > 5–20 × ULN), kidney function tests (RIFLE criteria SrCrea × 2) [14], cytopenia (neutropenia if N ≤ 1500/mmc), and hypernatremia (Na ≥ 146 mmol/L).

### Statistical analysis

Descriptive statistics were used to summarise demographic characteristics, infection types, microbial data, and treatment outcomes. Continuous variables were presented as means ± standard deviations or median ± interquartile range, while categorical variables were expressed as frequencies and percentages. Statistical analysis was conducted using SPSS version 30.0 (IBM Corp, Armonk, NY).

## Results

A total of 50 paediatric patients were included, with a median age of 5.93 years (IQR 9.71) (Table 2). Most patients (78%) were older than 1 year, while 6% were neonates. The sex distribution was balanced (54% male). Comorbidities were present in 80% of the cohort, including oncological conditions (30%), congenital heart defects (18%), genetic syndromes, prematurity, cystic fibrosis and chronic neurological disorders. Immunosuppressive conditions (either primary or treatment-related) were observed in 56% of cases.

**Table 2:** Demographic and clinical characteristics of the study population

Variable		Value
Number of patients *N*	*-*	50
Median age Y (IQR)	*-*	5.93 (9.71)
Age categories *N* (%)	*Neonatal age* *1–12 months* *1–10 years* > *10 years*	3 (6%) 8 (16%) 21 (42%) 18 (36%)
Sex *N* (%)	*Male* *Female*	27 (54%) 23 (46%)
Comorbidities *N* (%)	*-*	40 (80%)
Type of comorbidities *N* (%)	*Oncological diseases* *CHD* *Prematurity* *Others*	15 (30%) 9 (18%) 2 (4%) 14 (28%)
Immunosuppression *N* (%)	*-*	28 (56%)
Type of medical devices *N* (%)	*CVC* *ET* *ECMO* *Surgical drain* *PEG*	47 (94%) 11 (22%) 6 (12%) 5 (10%) 3 (6%)
ICU admission *N* (%)	-	12 (24%)
Antibiotic therapy in the previous 30 days *N* (%)	**-**	41 (82%)
Positive microbiological cultures *N* (%)	-	45 (90%)
Fosfomycin MIC *N* (%)	MIC < 16 mg/L MIC < 32 mg/L NA	16 (51.6%) 15 (48.4%) 14
Fosfomycin mean daily dose (mg/kg/day) (IQR)	**-**	288 (92-320)
Fosfomycin therapy duration, *N* (IQR)	Days	12 (9)

Most patients (94%) had CVCs, and many required additional invasive support such as ET (22%), ECMO (12%), surgical drainage (10%) or PEG (6%). Prior antibiotic treatment within the previous 30 days was recorded in 82% of cases.

Infections treated included sepsis (54%), osteoarticular infections (12%), skin and soft tissue infections (8%), and other severe infections involving the lungs (14%), central nervous system (4%), or abdomen (6%). IV fosfomycin was used exclusively in combination therapy and was introduced as rescue therapy, particularly in cases of failure of first-line treatments, confirmed or suspected MDR pathogens, and critical clinical situations requiring compassionate use.

Microbiological confirmation was achieved in 90% of cases, with positive blood cultures in 35.6%, cultures from infection sites in 48.9% and colonising swabs in 15.6% (Table 3).

**Table 3:** Clinical and microbiological characteristics of the study population

		Gram negative	Gram positive	No microbiological identification
Number of cases *N* (%)		24 (48%)	21 (42%)	5 (10%)
Type of infection *N* (%)	Sepsis SSTIs Osteoarticular infections Others (pneumoniae, SNC infections, intra-abdominal infections)	15 (62.5%) 2 (8.3%) 1 (4.2%) 6 (25%)	11 (52.3%) 1 (4.7%) 4 (19%) 5 (23.8%)	1 (20%) 1 (20%) 0 (0%) 3 (60%)
Pathogen *N* (%)	*Enterobacteriacee* *P. aeruginosa* *S.aureus* *CoNS*	19 (79.2%) 5 (20.8%) - -	- - 11 (52.3%) 7 (33.3%)	- - - -
Resistance *N* (%)		15 (62.5%)	11 (52.3%)	-
Type of resistance *N* (%)	*ESBL* *KPC* *MBL* *P.aeruginosa XDR* *Oxa R* *XDR*	2 (8.3%) 1 (4.2%) 9 (37.5%) 1 (4.2%) - 1 (4.2%)	- - - - 11 (52.3%)	- - - - -
Combination therapy *N* (%)	Glycopeptide Aminoglycoside Carbapenem Beta-lactam Anti-MRSA other than glycopeptide	6 (25%) 7 (29.1%) 14 (58.3%) 10 (41.6%) 3 (12.5%)	6 (28.5%) 0 (0%) 5 (23.8%) 10(47.6%) 9 (42.8%)	0 (0%) 0 (0%) 3 (60%) 2 (40%) 2 (40%)
Outcome *N* (%)	Recovery Relapse Death	18 (75%) 1 (4.1%) 5 (20.8%)	18 (85.7%) 3 (14.2%) 0 (0%)	5 (100%) 0 (0%) 0 (0%)

*Gram*-negative organisms accounted for 53.4% of microbiologically confirmed infections. Enterobacterales were the most frequent, including ESBL and carbapenemase-producing strains. A total of 62.5% of identified *Gram*-negative strains were resistant to first-line therapies. The pathogens included 2 ESBL-producing Enterobacterales (8.3%), 11 carbapenemase-producing Enterobacterales (45.8%), an extensively drug-resistant *Pseudomonas aeruginosa* (4.2%) and one extensively drug-resistant *Enterobacter cloacae* (XDR E. cloacae) (4.2%). Fosfomycin was combined with carbapenems, aminoglycosides, or targeted agents based on susceptibility testing. Fosfomycin was used as a second-line agent in 60.87% of these cases, third-line in 26.1%, and fourth-line in 8.7%. The overall cure rate for *Gram*-negative infections was 75%, with a 20.8% mortality and a 4.1% treatment failure.

*Gram*-positive infections accounted for 46.6% of microbiological diagnoses and were primarily caused by *Staphylococcus aureus* (52.3%), coagulase-negative staphylococci (33.3%), 2 *Streptococcus* spp. and 1 *E. faecium*. A total of 33.3% of all *Gram*-positive isolates were Oxacillin-resistant. Fosfomycin was mainly combined with glycopeptides or other MRSA-active agents (as daptomycin or linezolid). It was introduced as second-line therapy in 54.55% of these cases and as third- or fourth-line in the remainder (36.36% and 9.09%). MIC data for fosfomycin were available for 31 cases: 16 had MIC < 16 mg/L (51.6%), and 15 had MIC ≤ 32 mg/L (48.4%). Fourteen cases lacked MIC data. The average duration of IV fosfomycin therapy was 12 days (IQR = 9).

Among patients with bacteremia and indwelling central venous catheters, catheter removal occurred in 71.4% of Gram-negative bloodstream infections, in 100% of *Staphylococcus aureus* bacteremia, and in 40% of coagulase-negative staphylococcal bacteremia. In the remaining Gram-negative cases, catheter removal was not feasible due to the severity of the patients’ clinical conditions. In coagulase-negative staphylococcal bacteremia, catheters that were not removed were successfully managed with antibiotic lock therapy. The mean time to catheter removal was 3.2 days from blood culture sampling.

Therapeutic failure, occurred in 28% of patients. There were five deaths (10%), all related to the infectious episode and caused by *Gram*-negative infections, while two additional deaths were attributed to underlying chronic illness and occurred more than 12 weeks after the infectious episode. Notably, no deaths were observed in cases involving *Gram*-positive infections.

Outcomes varied by infection type: circulatory infections, defined as bloodstream infections (primary bacteremia, catheter-related bloodstream infections, and one case associated with an infected biofilm on Berlin Heart cannulas), had a recovery rate of 80.8%, with 11.5% relapsing and 7.7% dying; no cases of infective endocarditis were observed. SSTIs had a 33.3% mortality rate (1 death in 3 patients), while osteoarticular infections had a 100% cure rate. Twelve patients required intensive care unit admission, equally distributed between *Gram*-positive and *Gram*-negative infections; in two cases, no pathogen was identified.

Pre-treatment labs showed neutropenia < 0.5 × 10^9^/L in 12 patients (24%) and thrombocytopenia < 50 × 10^9^/L in the same number. Severe anaemia (Hb < 7 g/dL) was reported in 3/46 cases (6.5%). The median sodium value at the beginning of the therapy was 137 mmol/L, and none of the patients in the population developed hypernatremia during therapy. Three patients had pre-existing acute kidney injury (AKI) and had their fosfomycin dosage adjusted, but this was not attributed to fosfomycin-induced nephrotoxicity. In the study population, liver toxicity, defined as ALT/AST > 3 × the upper limit of normal (ULN), was observed in 5 out of 50 cases (10%). One patient developed grade 1 hepatotoxicity, three patients developed grade 2, and only one experienced grade 3 hepatotoxicity, which did not require discontinuation of therapy.

## Discussion

This study evaluated the use of IV fosfomycin in a complex paediatric population with severe infections, frequently caused by MDR pathogens and often refractory to standard treatments. Patients were notably fragile, with a high burden of comorbidities, immunosuppression, and dependence on invasive support.

Fosfomycin was used in all cases as part of combination therapies, typically with carbapenems, glycopeptides, or daptomycin or linezolid and aminoglycosides. Its broad spectrum, bactericidal effect and synergistic properties supported its selection in critical settings, especially following the failure of standard antibiotics or where other therapeutic options were limited. In patients without microbiological evidence of an isolated pathogen, fosfomycin was used as part of a calculated combination therapy, such as carbapenems plus an anti-MRSA agent (e.g. carbapenems + linezolid/daptomycin + fosfomycin).

Fosfomycin showed promising outcomes in infections caused by MDR *Gram*-negative bacteria, particularly ESBL- and carbapenemase-producing Enterobacterales. IV fosfomycin was used as a rescue therapy for two infections caused by XDR G- pathogens, specifically in the treatment of a skin ulcer due to *Enterobacter cloacae* and a meningoencephalitis caused by *Pseudomonas aeruginosa*. Its combination with other agents such as carbapenems or aminoglycosides yielded an overall cure rate of 75% with a survival rate of 90%, suggesting a potential clinical benefit even in severe systemic infections. Though less commonly reported, fosfomycin also demonstrated efficacy against *Oxacillin-resistant Gram-positive pathogens*, including MRSA and coagulase-negative staphylococci. Used in combination with glycopeptides or MRSA-specific drugs, it provided favourable outcomes without associated mortality, suggesting a potential role in treating resistant *Gram*-positive infections. A small number of patients (*n* = 5) had culture-negative infections but were included based on severe clinical and radiological evidence of invasive infection and prior exposure to broad-spectrum antibiotics.

The deaths reported in this study should be interpreted in the context of the patients’ underlying conditions. Five deaths were directly attributable to infection: one due to septic shock, one due to early-onset neonatal sepsis (EONS) in a severely preterm infant, one due to severe meningoencephalitis, and two due to multi-organ failure (MOF) secondary to infection. One of the MOF-related deaths, which occurred in a cardiac intensive care patient patient with a cutaneous ulcer infected with *Enterobacter cloacae*, contributed to the lower survival rate observed among patients with SSTIs, a group comprising only three individuals in the study population. Osteoarticular infections, however, had a 100% cure rate. The remaining two deaths were unrelated to the infectious episodes, with one occurring due to a relapse of acute myeloid leukaemia (AML) post-transplantation and the other due to congenital heart disease.

The most common side effects of fosfomycin IV are gastrointestinal symptoms, allergic reactions, hepatotoxicity with elevated transaminases, nephrotoxicity, and cytopenia. Since fosfomycin is predominantly excreted unchanged in the urine, its dosage should be adjusted based on renal function. As presented in the results, three patients had acute kidney injury (AKI) prior to the initiation of fosfomycin therapy. In these cases, fosfomycin was chosen over aminoglycosides to retain bactericidal efficacy while aiming to preserve renal function; the drug was also administered at a dose adjusted to kidney function. Fosfomycin infusion may also cause electrolyte imbalances, as the solution contains fosfomycin disodium (1 g of fosfomycin contains 0.32 g of Na). In this study, IV fosfomycin demonstrated good safety and tolerability, even among fragile patients with comorbidities. Notably, two severely preterm neonates (weighing 570 g and 670 g) received IV fosfomycin without significant side effects. In this regard, Obiero’s study on neonatal sepsis confirmed that fosfomycin had no impact on blood sodium levels or gastrointestinal function in neonates [9]. Moreover, the observed toxicity in the study population is difficult to attribute to fosfomycin therapy, as these patients had pre-existing critical clinical conditions. Additionally, the retrospective nature of the study introduces potential confounding factors that complicate the evaluation.

Fosfomycin is traditionally used to treat uncomplicated urinary tract infections (UTIs), but its use in treating severe, multidrug-resistant infections has become more prominent in recent years [15–18]. In 2020, the EMA approved IV fosfomycin for a range of severe infections, including complicated UTIs, infective endocarditis, bone and joint infections, hospital-acquired and ventilator-associated pneumonia, complicated SSTIs, bacterial meningitis, complicated intra-abdominal infections, and bacteremia [19–21]. Its use in paediatrics, however, remains limited. Our findings are consistent with recent studies that demonstrated the efficacy and safety of IV fosfomycin in paediatric populations, including for pneumonia, sepsis, and osteoarticular infections [4, 6, 21–25]. Notably, our study adds to the evidence base by focusing on a more complex population, often excluded from clinical trials, thereby reinforcing the utility of fosfomycin in real-world high-risk settings.

As a retrospective, single-centre study, our findings are subject to several limitations, including the heterogeneity of infections, pathogens, and patient characteristics, as well as the absence of standardised microbiological monitoring during treatment. In addition, the small number of patients in some indication subgroups limits the robustness of subgroup analyses. Furthermore, because all patients received combination therapy and no control group without fosfomycin was available, it was not possible to perform an objective assessment of the independent efficacy of fosfomycin. Finally, given the retrospective design and the variability in treatment duration and observation periods, it was not feasible to evaluate the components of the composite endpoint at predefined fixed time points (e.g. 30-day mortality, 90-day relapse, or early clinical improvement), as this would have resulted in non-uniform follow-up and introduced additional sources of bias.

## Conclusions

Despite these challenges, the use of IV fosfomycin in combination therapy appeared to be a reliable therapeutic option in managing difficult-to-treat infections in paediatric settings where standard antibiotics may be insufficient [26, 27].

Its efficacy was observed in both *Gram*-negative and *Gram*-positive infections, with good clinical responses even in cases involving ESBL- and carbapenemase-producing Enterobacterales or MDR *Gram*-positive. IV fosfomycin has demonstrated good tolerability, with minimal adverse effects even in patients with significant comorbidities, immunosuppression, and exposure to intensive care interventions.

Further studies, particularly those involving larger cohorts and controlled trials in paediatric populations, are needed to define better the role of IV fosfomycin in paediatric infection management, optimise dosing strategies, and assess long-term outcomes. 

## Data Availability

The datasets used and/or analysed during the current study are available upon reasonable request from the corresponding author.

## Abbreviations

UDP: Uridine diphosphate
G -: Gram-negative
G +: Gram-positive
MDR: Multidrug-resistant
UTIs: Urinary tract infections
CNS: Central nervous system
SSTIs: Skin and soft tissue infections
IV: Intravenous
CVC: Central venous catheters
ET: Endotracheal tubes
ECMO: Extracorporeal membrane oxygenation
PEG: Percutaneous endoscopic gastrostomy
ESBL: Extended-spectrum beta-lactamase
CRE: Carbapenem-resistant Enterobacteriaceae
MICs: Minimum inhibitory concentrations
AST: Aspartate aminotransferase
ALT: Alanine aminotransferase
ULN: Upper limit of normal
RIFLE: Risk – injury – failure – loss – end-stage kidney disease
SPSS: Statistical package for the social sciences
IQR: Interquartile range
XDR: Extensively drug resistant
MRSA: Methicillin resistant *Staphylococcus aureus*
AKI: Acute kidney injury
EONS: Early-onset neonatal sepsis
MOF: Multi-organ failure
AML: Acute myeloid leukaemia
EMA: European medicines agency
